# Is COVID-19 All That Glitters?

**DOI:** 10.3390/jcm12072552

**Published:** 2023-03-28

**Authors:** Salvatore Spampinato, Maurizio Di Marco, Luciano Mammolito, Alessia Scarfia, Maurizio Valastro, Stefania Di Mauro, Giosiana Bosco, Francesco Purrello, Salvatore Piro

**Affiliations:** 1 Department of Clinical and Experimental Medicine, Internal Medicine, Garibaldi-Nesima Hospital, University of Catania, 95122 Catania, Italy; 2 Radiodiagnostic Unit, Highly Specialized Hospital of National Importance “Garibaldi”, 95122 Catania, Italy

**Keywords:** COVID-19, pandemic, SARS-CoV-2 infection, MIS-C, large vessels vasculitis, Takayasu arteritis

## Abstract

Over the last three years, the Coronavirus-19 disease has been a global health emergency, playing a primary role in the international scientific community. Clinical activity and scientific research have concentrated their efforts on facing the pandemic, allowing the description of novel pathologies correlated to Severe Acute Respiratory Syndrome Coronavirus 2 (SARS-CoV-2), such as the Multisystemic Inflammatory Syndrome in Children and Adults (MIS-C, MIS-A). Conversely, this shift of attention to COVID-19 disease and its complications could, in some cases, have delayed and underestimated the diagnosis of diseases not associated with SARS-CoV-2, including rare diseases. Here we describe the diagnostic process that led to the definition of a rare vasculitis in a young woman with a recent clinical history of SARS-CoV-2.

## 1. Introduction

COVID-19 disease has dramatically affected the entire world, as demonstrated by the 663,64 million proven-infected subjects and 6.7 million deaths since the beginning of the pandemic [1]. The efforts conducted by the National Health System and the scientific community were pivotal in dealing with this health emergency and containing the pandemic. Indeed, SARS-CoV-2 infection effectively represented a novel diagnostic and therapeutic challenge. In this context, novel nosological entities have been described, often represented by COVID-19 complications. Multisystemic Inflammatory Syndrome in Children (MIS-C) and Multisystemic Inflammatory Syndrome in Adults (MIS-A) are rare clinical conditions characterized by an abnormal inflammatory response observed in patients with recent SARS-CoV-2 infection. Fever, elevated inflammatory markers, and multiorgan dysfunctions (heart, gastrointestinal tract, respiratory system, and skin) characterize MIS-C/A [2,3]. Furthermore, this syndrome can have heterogeneous clinical manifestations in common with Kawasaki Disease (KD), Macrophage Activation Syndrome (MAS), and Toxic Shock Syndrome (TSS) [4]. The CDC and the WHO diagnostic criteria for MIS-C establish that alternative diagnoses, including vasculitis, must be excluded [5,6]. This complex diagnostic process relies on clinical judgment. To date, the error of associating everything with COVID-19 is concrete, risking underestimating or deleting rare disease diagnoses.

## 2. Case Description

An 18-year-old woman was admitted to our hospital’s Emergency Department (ED) because of a history of intermittent fever, fatigue, and tachycardia for one month. These symptoms started after a positive SARS-CoV-2 molecular swab test. She reported no past medical or surgical history or allergies. Medication consumption was denied, and she had declined the COVID-19 vaccine. The patient reported that during the SARS-CoV-2 infection, she had a fever, mild exertional dyspnea, “brain fog”, tachycardia, and loss of appetite. At that time, the general practitioner (GP) recommended antimicrobial (Clarithromycin 250 mg os BID) and corticosteroid (Prednisone 25 mg os QD) therapy for six days with transient clinical benefit. After a negative molecular swab test, the fever relapsed in an intermittent pattern reaching 39 °C. According to the GP’s recommendation, blood tests showed elevated C-reactive protein (CRP) and erythrocyte sedimentation rate (ESR) with no leukocytosis. Because of the one-month persistent symptoms since the SARS-CoV-2 infection, the patient was admitted to the ED of our hospital and then transferred to our Internal Medicine Department with the diagnosis of “fever and fatigue after a recent SARS-CoV-2 infection”.

On admission, she reported severe fatigue and weight loss over the previous two months (>5% body weight). The molecular swab test for SARS-CoV-2 was negative. A physical examination showed tachycardia (a heart rate of 110 bpm), no absent or weak peripheral pulses, normal conjunctiva, no skin rash, no superficial lymphadenopathy, and no joint redness or swelling. Sinus tachycardia (a heart rate of 120 bpm) was recorded by an electrocardiogram without other findings. Thus, routine laboratory tests and the temperature curve were performed. Blood tests showed elevated CRP/ESR, hyperfibrinogenemia, prolonged activated partial thromboplastin time (aPTT), and international normalized ratio (INR) (Table 1). The temperature curve recorded daily fever in the evening (peak 38.5 °C) without any signs or symptoms.

Supported by the clinical and laboratory findings, the differential diagnosis for Fever of Unknown Origin (FUO) was started following a recently available flowchart [7].

According to this algorithm, we could rule out various factors:

- Travel abroad, contact with animals, and in-depth laboratory tests excluded any specific infectious etiology such as endocarditis, zoonoses (e.g., brucellosis, Lyme disease, and rickettsioses), typhoidal and nontyphoidal salmonella, chlamydia pneumoniae, mycoplasma pneumoniae, cytomegalovirus (CMV), Epstein–Barr virus (EBV), human immunodeficiency virus (HIV), syphilis, and mycobacterial infection;
- Lymphoproliferative disorders were excluded (no lymphadenopathy, and the peripheral blood smear, white blood cell count, and lactate dehydrogenase levels were normal);
- At the liver and spleen ultrasound examinations, no pathological findings were found;
- Rheumatological diseases were excluded (skin rash, mucous involvement, and redness and swelling of the joints were not observed in our patient; ANA, ENA, p-ANCA, c-ANCA, and antiphospholipid antibody testing were negative).

In the current historical context, the diagnostic workup could have found a possible conclusion with the diagnosis of MIS-C. In our patient, the persistent fever, the “brain fog”, the elevated CRP/ESR and the prolonged aPTT/INR associated with the recent SARS-CoV-2 infection supported the MIS-C hypothesis but did not fully meet the CDC and the WHO criteria for the diagnosis. For this reason, it was mandatory to continue the diagnostic workup to rule out other FUO-possible etiologies.

**Figure 1:**
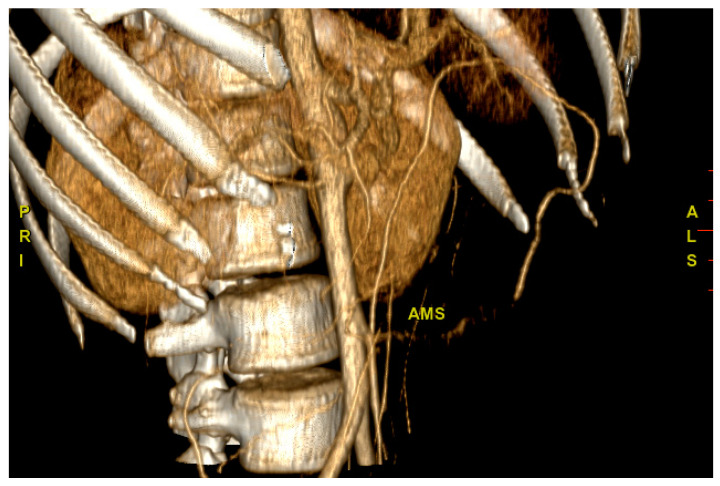
CT scan of the abdomen. Proximal occlusion of the superior mesenteric artery (AMS).

**Figure 2:**
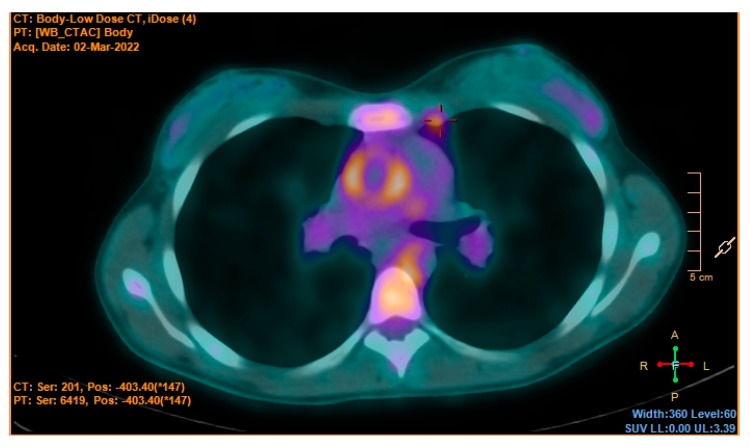
PET-TC. Increased metabolic activity of the ascendent aorta.

**Figure 3:**
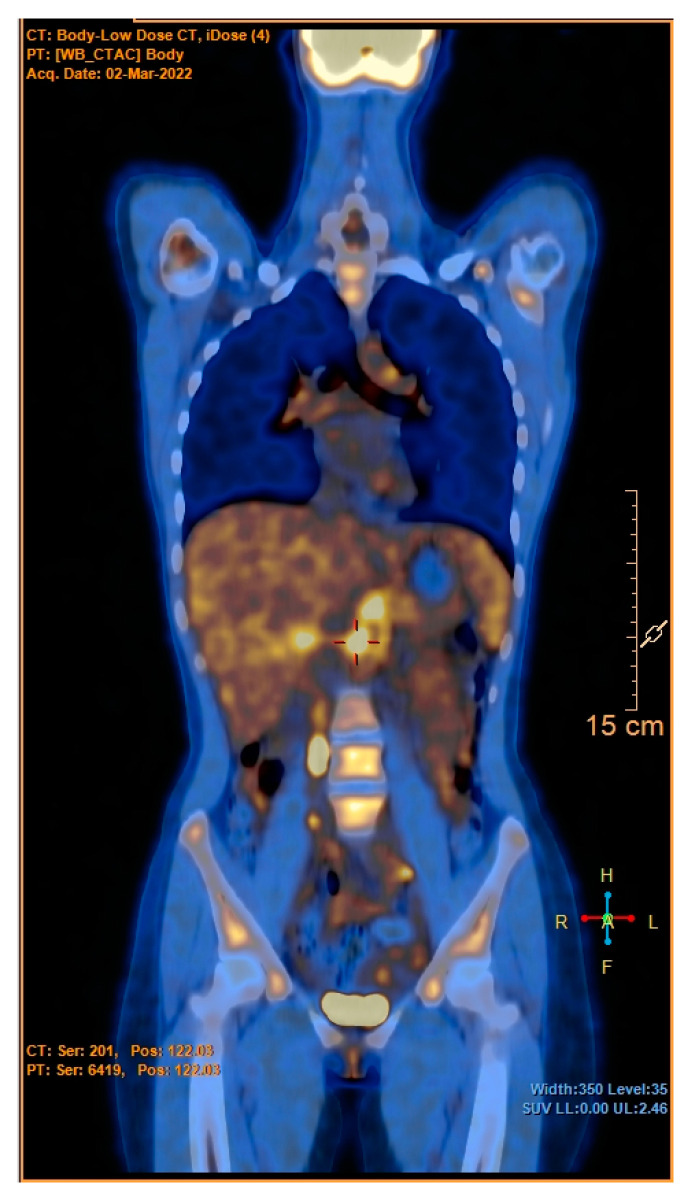
PET-CT. Increased metabolic activity of the suprarenal abdominal aorta.

According to the flow chart, CT scans of the chest and the abdomen were the next steps. This decision was carefully evaluated, balancing the risks and benefits in consideration of the patient’s young age. We decided to perform the CT scan that showed an extensive wall thickening of the aortic arch, the abdominal aorta and its main branches, and proximal occlusion of the superior mesenteric artery (Figure 1). A PET-CT was then performed and showed abnormal metabolic activity of the aortic arch, the ascending and descending thoracic aorta, and the abdominal aorta (Figure 2 and Figure 3). These findings were suggestive of large-vessel arteritis.

According to the EULAR/PRINTO/PRES for childhood Takayasu Arteritis, the diagnosis of Takayasu arteritis (TA) was made [8]. On a rheumatologist’s recommendation, corticosteroid therapy at a high dose (Prednisone 1 mg/kg os QD) was administered. The patient reported clinical benefit; moreover, the near normalization of the inflammatory markers after seven days of therapy was observed. She was then discharged home with further medical management.

## 3. Discussion

Takayasu arteritis is a chronic granulomatous panarteritis, mainly observed in subjects under 40, that affects the aorta, its major branches, and the pulmonary arteries. The chronic inflammatory process causes wall thickening with subsequent formation of stenoses, occlusions, and/or aneurysms of the affected vessels. Aspecific constitutional symptoms such as fever, anorexia, weight loss, and myalgias are often reported by affected patients. As the vascular damage progresses, symptoms and signs due to peripheral perfusion deficit are observed: claudication of the lower limbs, reduced or eliminated peripheral pulses, vascular bruits, arterial hypertension, carotidynia, abdominal pain, heart failure, transient ischemic attack, and stroke [9]. The undefined and non-specific manifestations of this rare vasculitis in earlier phases represent a diagnostic challenge for the clinician. Often the diagnosis is reached after months or years when the most critical disease consequences, for instance, vascular stenoses or tissue ischemia, are already evident [10].

Why were we falling into the error of associating the young patient’s clinical presentation with SARS-CoV-2? We believe that the recent viral infection and the temporal coincidence of clinical symptom manifestation played a decisive role in this incorrect evaluation. Indeed, the CDC diagnostic criteria for MIS-C indicate a temporal window of four weeks between symptom onset and SARS-CoV-2 infection, which is the same as that observed in our patient. Another parameter that led us to focus our attention on this COVID-19 complication was the presence of a highly undefined clinical presentation. Briefly, intermittent fever, asthenia, the elevation of systemic inflammation indices, and coagulation alteration are signs and symptoms in common with vasculitis such as MIS-C; however, the time coincidence with the recent viral infection and the tendency to focus our attention on SARS-CoV-2 infection in this precise historic context led us to mainly take into account MIS-C. This definitive diagnosis would have been a serious mistake with significant consequences for the health of our young patient. It has been reported in the literature the clinical case of a 35-year-old woman with heart failure whose symptoms overlapped with the heterogeneous manifestations of COVID-19. These similarities and the change of priorities during the pandemic period delayed the diagnosis of acute heart failure determining significant consequences on the patient’s clinical condition [11].

Therefore, what lessons can we learn from this clinical experience? The pandemic’s rough socio-economic impact demonstrates how SARS-CoV-2 infection is indisputably the protagonist of our historical context. The redistribution of resources in health systems and the reduction of hospitalizations during the pandemic caused serious repercussions for medical care for both chronic and acute diseases not associated with SARS-CoV-2 [12]. We believe that being aware and remembering what happened will lead us, in the future, to evaluate the patient without preconceptions, thus defining priorities and following the diagnostic-therapeutic process according to scientific evidence.

In this case report, a reason for reflection is also the diagnosis of TA one month after the SARS-CoV-2 infection considering the already-known relationship between viral infections and autoimmune disease [13]. Respiratory tract viruses, including parainfluenza virus (PIV), influenza, respiratory syncytial virus (RSV), and adenovirus, are commonly associated with Henoch–Schönlein Purpura or IgA vasculitis, a type of small vessel vasculitis [14]. Hepatitis C virus (HCV) is the most frequent cause of cryoglobulinemic vasculitis, a vasculitis of small and medium vessels due to the deposition of mixed cryoglobulins and immunocomplexes [15]. Polyarteritis nodosa has been linked to hepatitis B virus infection with the formation of multiple microaneurysms as well as the corkscrewing of medium-sized arteries, most commonly the renal arteries, visible at angiography or biopsy [16]. Still, the pathogenetic mechanism that links viral infection and autoimmunity is not clear enough. Available data suggest that viruses are considered major environmental factors that trigger the autoimmune phenomena in genetically susceptible individuals through multiple mechanisms, mainly by molecular mimicry [13]. According to these data and the increasing evidence suggesting an association between the SARS-CoV-2 infection and autoimmunity [17], it is possible to hypothesize the presence of a link between SARS-CoV-2 infection and vasculitis. Indeed, during the pandemic, several SARS-CoV-2-associated Takayasu arteritis cases in young patients of a similar age to our clinical case have been described [18,19,20]. Mendes et al. described the case report of a 19-year-old woman who presented fatigue and malaise one month after a SARS-CoV-2 infection. Adequate diagnostic workup led to a Takayasu arteritis diagnosis, as shown by the concentric and diffuse thickening of the thoracic and abdominal aorta with a CT scan, similar to our case report [18]. To date, nevertheless, there is a limited number of literature studies supporting the existence of a causal link; thus, it is not possible to exclude that the association between SARS-CoV-2 and Takayasu arteritis was casual in reported clinical cases.

## 4. Conclusions

Considering the pandemic period, the clinical presentation of our patient could have been attributed to a SARS-CoV-2 infection complication in the emergent nosography entity called MIS-C. In this case, we think that it was fundamental not to have underestimated the young patient’s illness, in a period where there is the risk of associating everything with COVID-19. However, are we sure that SARS-CoV-2 is totally innocent? The link between viral infections and vasculitis has already been described in the literature and we cannot exclude it in the examined case in consideration of the temporal coincidence. However, in this case, the correlation between the infection and the autoimmune disease is not easy to evaluate. In conclusion, it is mandatory to suspect that not all that glitters is COVID-19.

## Figures and Tables

**Table 1:** Clinical and laboratory findings obtained at admission.

Clinical and Laboratory Findings	Value	Normal Range
Pulse	110 beats/min	60–100
Blood pressure	128/80 mmHg	110–131/64–83
Temperature	38.5 Celsius	36.3–37.6
Respiratory rate	18 breaths/minute	12–20
BMI	18.6 Kg/m^2^	18.5–24.9
Hemoglobin	9.5 g/dL	12.0–15.5
Mean corpuscular volume	73.7 fL	80.4–95.9
White blood cells	7.60 × 10^3^/µL	4.10–11–20
Platelets	551 × 10^3^/µL	159–388
Creatinine	0.51 mg/dL	0.60–1.30
Aspartate aminotransferase	19 UI/L	5–34
Alanine aminotransferase	6 UI/L	0–55
Creatine phosphokinase	29 UI/L	29.0–200.0
Iron	15 µg/dL	50–170
Ferritin	338 ng/mL	5–204
C-reactive protein	12.59 mg/dL	0.01–0–50
Procalcitonin	0.10 µg/L	<0.05
ESR	113 mm	0–15
Fibrinogen	671 mg/dL	200–450
Prothrombin activity	45%	80–120
Partial thromboplastin time	39 SEC	25–35
INR	1.70	0.90–1.10
TSH	1.70 microµ/mL	0.35–4.94
Thyroxine	1.02 ng/dL	0.70–1.48

BMI: Body Mass Index. MCV: Mean Corpuscular Volume. ESR: Erythrocyte Sedimentation Rate. INR: International Normalized Ratio. TSH: Thyroid Stimulating Hormone.

## Data Availability

Not applicable.
